# Efficacy of Kinesio taping in post operative sequalae after surgical removal of mandibular third molars: a split mouth randomized control study

**DOI:** 10.1186/s12903-023-03631-6

**Published:** 2023-12-04

**Authors:** Sneha Patil, Rajanikanth K., Nitin Bhola

**Affiliations:** Department of Oral and Maxillofacial Surgery, Sharad Pawar Dental College and Hospital, Datta Meghe Institute of Higher Education and Research (Deemed to Be University), DMIMS Campus, Radhikabai PG Hostel, F-27, Sawangi (M), Wardha, Maharashtra 442001 India

**Keywords:** Kinesio taping, Kinesiologic tape, Third molar, Transalveolar extraction

## Abstract

**Background:**

The surgical removal of impacted lower third molars produces a significant degree of trauma to the soft tissue and bony structures of the oral cavity, which can initiate considerable inflammatory reaction. Consequently, patient experiences pain, swelling, hemorrhage, nerve paraesthesia, limited mouth opening etc. The kinesiologic tape (KT) can help with blood and lymphatic circulation and has shown acceptable outcomes in reducing pain and in managing post-operative muscle spasm.

**Methodology:**

The study was aimed to compare the effects of kinesiologic tape on post operative pain, swelling and trismus following surgical removal of mandibular third molar when compared to control group. 15 patients with bilaterally impacted mandibular third molar were included in the split mouth study. One side was randomly assigned as Group A where patient’s face was subjected to Kinesio tape application post extraction. The other was Group B where Kinesio tape was not applied. After extraction each patient was evaluated in terms of post op pain, swelling and trismus in post-op day 1,2 and 7^th^.

**Results:**

The pain was found to be significantly less (*p* = 0.042) in group A when compared to group B on the first day. The mean pain was 5.07 in KT group and 6.20 in No KT group on day 2^nd^. Post OP Swelling was statistically significantly less (*p* < 0.01) in Group A on 2^nd^ & 3^rd^ day. The postoperative mouth opening was more from the 2^nd^ day in group A with mean of 26.07 mm and 20.33 mm in group B (*p* < 0.01).

**Conclusion:**

The kinesiologic taping originates from sports medicine, but can also used therapeutically for reducing post operative sequelae as demonstrated in our study. Kinesiologic tape (KT) enables patients to have a comfortable time post-operatively and helps to regain better quality of life.

**Trial registration:**

Registered in Clinical Trial Registry—India.

Registration number—CTRI/2021/05/033359, registration date – 04/05/2021.

## Introduction

Third molar transalveolar extractions are one of the most common procedures routinely performed in oral surgery. Subsequent to surgical removal of impacted lower third molars, there is significant degree of trauma to the soft tissue and bony structures in the oral cavity. Consequently, patients may experience numerous problems such as facial swelling, pain, hemorrhage, dry socket, nerve paraesthesia, and limited mouth opening [1, 2].

Amongst these postoperative outcomes of third molar surgeries- pain, difficulty in mouth opening and facial oedema are most common. An extensive research has been done in terms of post operative management of third molar surgeries, especially pertaining to pain, facial oedema and difficulty in mouth opening [1, 3]. The pain reaches its maximal level within 3–5 h after the cessation of anaesthetic effect, stays for 2–3 days, and then gradually fades until day 7. The swelling on the other hand, reaches its maximal level within 12–48 h and fades within 5–7 days [4, 5]. However, trismus purses until the fading away of pain and swelling.

KT is elastic therapeutic tape created by Dr. Kase in 1970s. KT was developed as an adjuvant treatment modality in sports medicine and it is primarily utilized to support injured soft tissues (muscles and joints). KT is believed to regulate blood and lymph flow along with removal of lymphatic fluid or haemorrhage congestions by elevating the skin and enabling fluids to shift from areas of higher pressure to lower pressure based on the direction of application. Moreover, KT influences muscular mechanoreceptors, which reduces pain and also facial oedema [4, 5].

In recent years, several researchers have successfully used KT following oral and maxillofacial surgery for temporomandibular problems, orthognathic therapy, mandibular fractures, and midface fractures. Although theory predicts that clinical advantages should follow, data to support this is scant. The aim of this study was to compare the effect of extra oral application of kinesiologic tape on post operative sequelae following surgical removal of mandibular third molar. The hypothesis for this study is that kinesiologic taping will reduce pain, edema and trismus after surgical removal of mandibular third molar.

### Objectives

1. To assess post-operative pain, swelling and trismus following third molar surgery after applying KT.
2. To assess post-operative pain, swelling and trismus following third molar surgery without applying KT.
3. To compare post-operative pain, swelling and trismus following third molar surgery in both the groups.

## Methodology

### Study design

The research was a prospective randomized control split-mouth non-blinded clinical study model to assess the impact of the KT on post-operative sequalae after surgical removal of mandibular third molar. With the cumulative 15 patients to be allocated for removal of bilateral involved mandibular third molar in near similar difficulty index 4-5 according to Pederson scale, justifying the extraction under local anaesthesia. The study was done under two groups, Group A – Test group (KT) one side of patient’s face, subjected to beige tape application. Group B – Control group (No KT) other side of patient’s face, will be the controlled group without application of KT.

### Sample setting

It was a hospital based experimental study in which the patient were allocated from the Out-Patient Department (OPD) of the “Department of Oral and Maxillofacial Surgery, Sharad Pawar Dental College, Sawangi, Wardha’.

### Study duration

November 2021- November 2022.

#### Methods: Assignment of interventions (for controlled trials)

- Allocation: Study population was randomized equally (n = 15) into two different groups (Test group & control group) using computer generated table of random numbers.
- Implementation: Independent observer
- Blinding (masking): Non blinding study
- Randomization: Simple using odd even method

#### Data collection, management, and analysis methods

Data collection methods: Patients reporting to OPD of “Oral and Maxillofacial Surgery department, Sharad Pawar Dental College”.

#### Ethics and dissemination

The study is approved from institutional ethical committee “Sharad Pawar Dental College” “[Ref. No- DMIMS(DU)/IEC/2020–21/9418].”

#### Declaration

The research was conducted under the Helsinki declaration 2013 and after approval by the guidelines prescribed by IEC of DMIMS DU.

### Sample size

Fifteen required in each groups.

### Sample size calculation

The sample size was calculated using the result of previous study of Gözlüklü Özgür et al. (2020) [4]. The following formula was used to calculate the sample size required for this study at 95% confidence interval and 80% power of the study.

$${\mathrm{n}}_{1}= \frac{{\left({\upsigma }_{1}^{2}+ {\upsigma }_{2}^{2}/\upkappa )({\mathrm{z}}_{1-\frac{\mathrm{\alpha }}{2}}+ {\mathrm{z}}_{1-\upbeta })\right.}^{2}}{{\Delta }^{2}}$$

$${\mathrm{n}}_{2}= \frac{{\left({\mathrm{k}}^{*} {\upsigma }_{1}^{2}+/{\upsigma }_{2}^{2})({\mathrm{z}}_{1-\frac{\mathrm{\alpha }}{2}}+ {\mathrm{z}}_{1-\upbeta })\right.}^{2}}{{\Delta }^{2}}$$

The notation for the formulae are:

${\mathrm{n}}_{1}$= sample size of group 1.

${\mathrm{n}}_{2}$= sample size of group 2.

σ_1_ = standard deviation of group 1.

σ_2_ = standard deviation of group 2.

∆ = difference in group means.

K = ratio = n_2_/n_1_.

z_(1-α/2)_ = two sided Z value (eg. Z = 1.96 for 95% confidence interval).

z_(1-β)_ = power.

The calculated sample size for this study was 15 per group keeping in mind the distribution was made equally, 15 subjects allotted in study group ( with KT ) and control group (No KT).

### Criterion for inclusion

1. Age of 18 years and above.
2. Bilateral impacted mandibular third molar in a near similar difficulty index.
3. A medical history devoid of any systemic pathological conditions.
4. A medical history devoid of any pharmacological therapy able to introduce variables into the experiment.

### Criterion for exclusion

1. Patients not willing to be a part of the study
2. Patients with uncontrolled systemic diseases.
3. Patients reluctant to follow instructions.

#### Surgical procedure

The surgical procedure involved two extractions for each patient, separated by a two-week interval. The procedure was performed by an operator under aseptic conditions following standard surgical protocols. Local anesthesia was administered using 2% lignocaine with 1:200,000 epinephrine, delivered by inferior alveolar nerve, lingual, and long buccal nerve block injections. The surgical incision was made using a No. 15 scalpel blade, and a mucoperiosteal flap was raised. Osteotomy was performed using round and fissured burs with sterile saline irrigation, and the tooth was extracted using an elevator or dental forceps. The socket was curetted, and the irregular bone borders of the alveolus were smoothed. Finally, the flap was repositioned and sutured using 3–0 silk.

#### Application of Kinesio tape

**Fig. 1:**
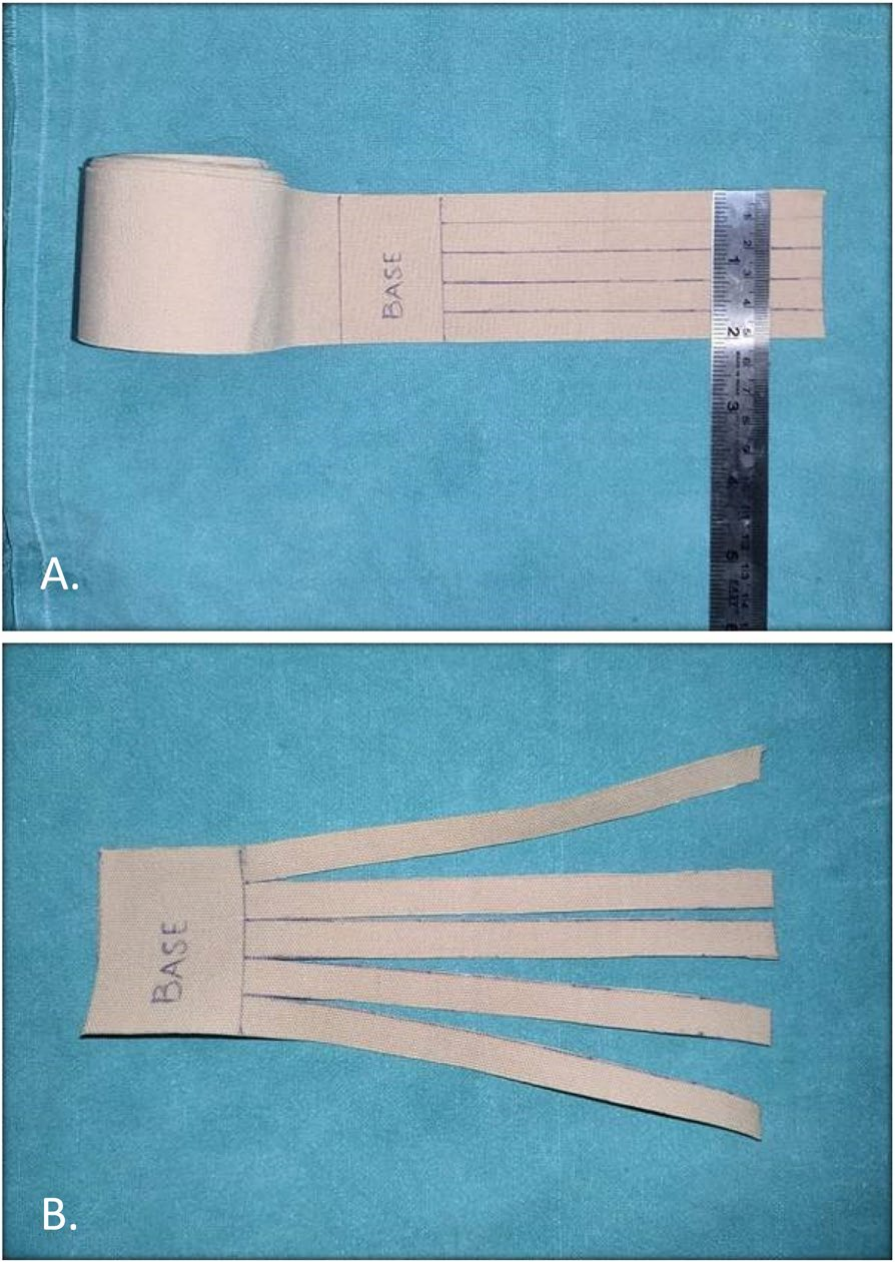
Preparation of Kinesio Taping. **A** The tape was cut into 5 equal strips, 1 cm in width and 18 cms in length. **B** Fan strips of Kinesio Tape

**Fig. 2:**
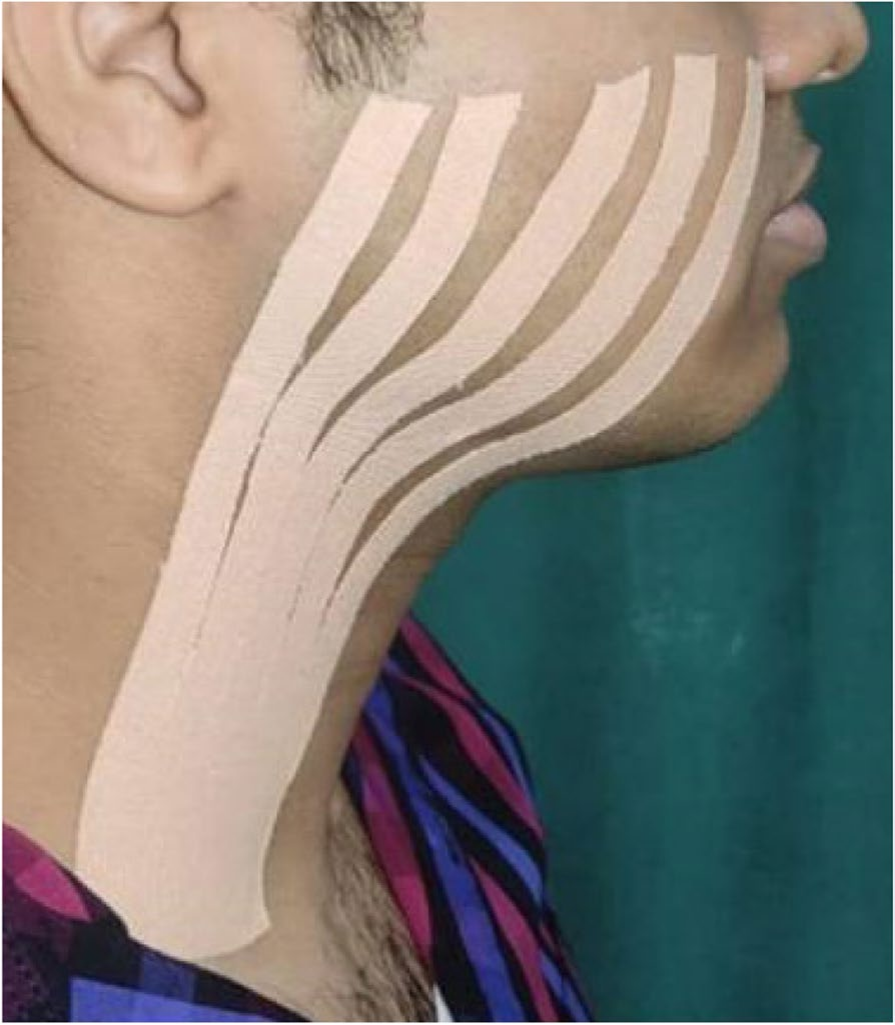
Application of Kinesio Tape in a patient

**Fig. 3:**
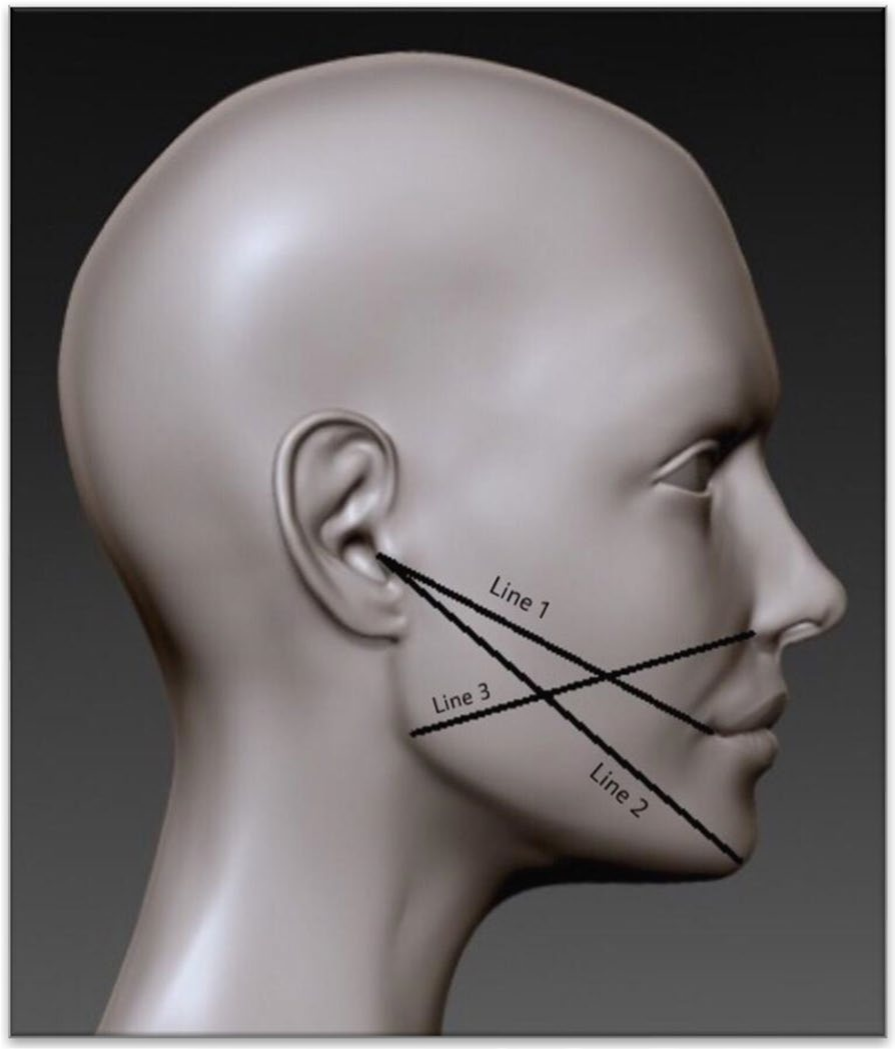
Three Reference lines for measurement of swelling

Following extraction, Kinesio Tape (KT) was applied to test group 1. The tape used was Kinesio Tex Gold Finger Print, with dimensions of 5 cm × 5 m. It was cut into five equal strips, each 1 cm in width and 18 cm in length, as shown in Fig. 1. The strips were placed between the clavicle and the tragus-commissure line (Fig. 2). The tape was applied every day for 7 days. It was changed once every morning. The patients were assessed for pain, swelling, and trismus. Pain was evaluated subjectively on postoperative days 1 (T1), 2 (T2), 3 (T3), and 7 (T7) using a Visual Analog Scale (VAS), with 0 indicating no pain and 10 indicating the worst pain. Facial swelling was measured using a measuring tape on preoperative and postoperative days 1, 2, 3, and 7. The measurements were taken in the three lines as shown in Fig. 3. The measurement lines were inspired from a study by Ana Carolina Heras et al. (2019) [1]. Line 1- the most posterior point of the tragus to the most lateral point of the lip commissure, Line 2- the most posterior point of the tragus to the soft tissue pogonion point, Line 3- the ala of the nose to the angle of the mandible. Maximal mouth opening that is maximum inter-incisal distance (IID) was measured post-operatively on first, second, third and seventh days by using vernier callipers.

The predictor variables in our study were age, gender, difficulty index of impacted tooth. However, the inclusion criteria was such that bias due to the above mentioned factors was not significant statistically. Outcome variables were post-operative pain, swelling, interincisal mouth opening. Same post operative medications were prescribed post operatively in both the groups therefore there were no major covariables in the study.

### Statistical analysis

Data so collected was tabulated in an excel sheet, under the guidance of statistician. The means and standard deviations of the measurements per group were used for statistical analysis (SPSS 22.00 for windows; SPSS inc, Chicago, USA). For each assessment point, data were statistically analyzed using one way ANOVA. Difference between two groups was determined using student t-test as well as chi square test and the level of significance was set at *p* < 0.05.

## Results

A total of 15 patients were evaluated in each category our study. The gender and age of the patient shows clinically insignificant bias as evaluated statistically in our study. There were 6 male patients and 9 female patients chosen for bilateral extraction of mandibular third molar. The mean age of the patients were noted to be 29.53 years.

**Fig. 4:**
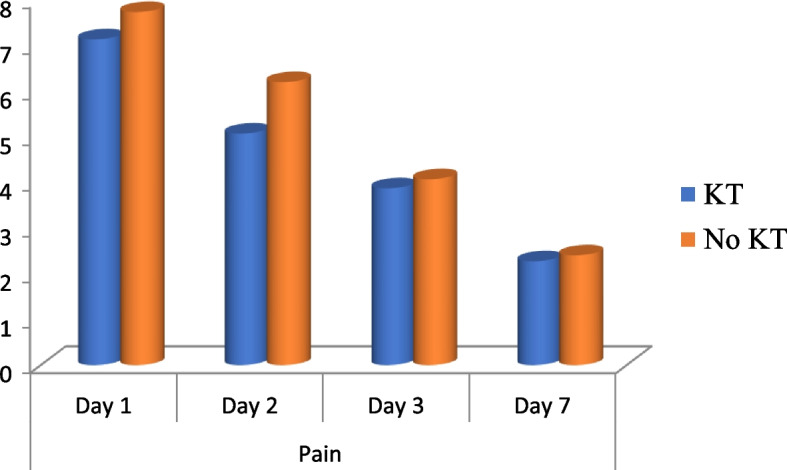
Comparison of pain at different intervals among the study groups

Statistical evaluation of the patient in whom third molar surgery was carried out in terms of pain, swelling and interincisal mouth opening in both taping group and control group. The pain was evaluated by VAS score. The mean of VAS score was noted to be 7.13 and 7.73 respectively in KT group and No KT group on first post-operative day. Pain was found to be significantly less (*p* = 0.042) in KT group when compared to the NO KT group on the first day. The mean pain was 5.07 in KT group and 6.20 in NO KT group on day 2^nd^ which implies patients with kinesio taping had more relief on the 2^nd^ day when compared to no application of kinesio tape (Fig. 4). Maximum pain is noted in the patients on the first and second day post-operatively which was significantly reduced in KT group (Table 1) shows it as an advantage of taping procedure. Overall pain was less in KT group when compared to the No KT group which was statistically significant(*p* < 0.01).

**Table 1:** Comparison of pain at different intervals among the study groups

Pain	KT		NO KT		t test	*P* value
	Mean	SD	Mean	SD		
Day 1	7.13	0.74	7.73	0.79	4.54	0.042*
Day 2	5.07	0.88	6.20	0.86	12.64	0.001**
Day 3	3.87	0.74	4.07	0.96	0.41	0.53
Day 7	2.27	0.46	2.40	0.74	0.35	0.56
Anova Test	13.57		13.09			
*P* value	< 0.01**		< 0.01**			

^*^: statistically significant, **: highly significant

**Fig. 5:**
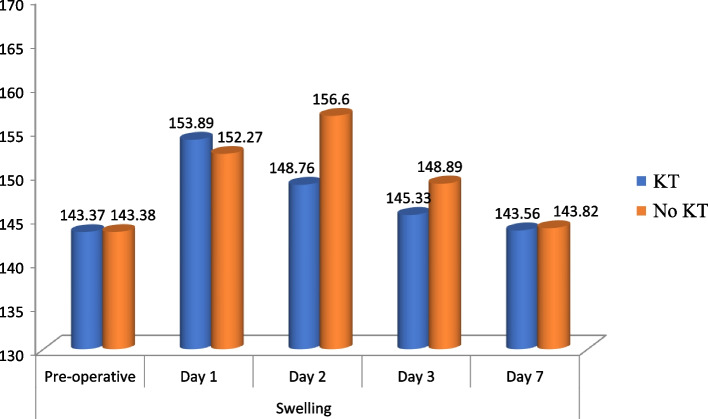
Comparison of swelling at different intervals among the study groups

The swelling in patients without taping was noted to increase till the 2^rd^ day (mean 156.60 mm) which is 9.23% increased signifying the inflammatory response of the patient when compared with pre-operative measurements. In KT group, the swelling increased to 7.31% (mean percentage) on 1^st^ day, 3.75% on day 2^nd^ and 1.36% on day 3^rd^ (Fig. 5). When KT group was compared to the No KT group in terms of extent of swelling, it was noted that the swelling was less with KT on 2^nd^ & 3^rd^ day which was highly significant statistically (*p* < 0.01) as shown in the Table 2. This signifies resolving of inflammatory response in KT and hence more comfort for the patient postoperatively. Overall swelling resolves by day 7^th^ in both the groups.

**Table 2:** Comparison of swelling at different intervals among the study groups

Swelling (mm)	KT		No KT		t test	*P* value
	Mean	SD	Mean	SD		
Day 1	153.89	6.87	152.27	6.04	4.84	0.23
Day 2	148.76	6.26	156.60	6.03	17.95	< 0.01**
Day 3	145.33	6.13	148.89	6.14	8.67	< 0.01**
Day 7	143.56	5.90	143.82	5.89	1.87	0.08
Anova Test	6.29		7.82			
*P* value	0.041*		0.009*			

^*^: statistically significant, **: highly significant

The postoperative mouth opening was more from the 2^nd^ day in KT group with mean of 26.07 mm and 20.33 mm in No KT group (*p* < 0.01). Trismus when noted on the 3^th^ day was also reduced with mean interincisal mouth opening of 29.13 mm in KT group and 23.20 mm when compared to the No KT group (Table 3). The recovery from trismus is directly related with reduction of pain and swelling from post-operative day 2^nd^ which is seen in patients and also verified by our results.

**Table 3:** Comparison of interincisal mouth opening among the study groups

Interincisal Mouth Opening (mm)	KT		NO KT		t test	*P* value
	Mean	SD	Mean	SD		
Pre-operative	34.27	1.94	34.53	1.64	0.17	0.69
Day 1	23.87	2.42	22.93	1.94	1.36	0.25
Day 2	26.07	1.98	20.33	2.55	47.19	< 0.01**
Day 3	29.13	1.46	23.20	3.26	41.50	< 0.01**
Day 7	33.67	1.88	33.20	1.89	0.46	0.50
Anova Test	8.91		9.27			
*P* value	0.008*		0.006*			

^*^: statistically significant, **: highly significant

## Discussion

Numerous methods have been tried and tested over time to minimalize the post operative complications associated with 3^rd^ molar removal. Kinesio taping has been used efficiently in orthopedic surgeries and sport medicine however its use in maxillofacial surgery has been limited. Therefore this study was designed with the aim to compare the effects of application of kinesiologic tape on post operative sequelae of surgical removal of mandibular third molars. We hypothesized that kinesiologic taping will reduce pain, edema and trismus post operatively. The motive behind this study was to find whether theoretically based findings about KT can be implemented clinically as well and we found that KT was effective in reducing post-operative pain, edema and trismus, particularly on second and third post-operative days.

One of the primary parameters, pain was reported to be significantly less in KT group when compared to the No KT group since the first day. The mean pain was 5.07 in taping group and 6.2 in control group on day 2 which implies KT group had lot more relief on the 2nd day when compared to NO KT. The results of this study suggest that kinesio taping is a superior modality to reduce the post operative pain in patient who has undergone third molar surgery. When Kinesio taping group was compared to the non-taping group to assess the extent of swelling, it was noted in our study that the swelling was less in taping group which was highly significant statistically when compared on the 2^nd^ day and 3^rd^ day. The swelling in patients without taping was noted to increase till the 2^rd^ day (mean 156.60 mm) which is 9.23% increased signifying the inflammatory response of the patient when compared with pre-operative measurements. In KT group, the swelling increased to 7.31% (mean percentage) on 1^st^ day, 3.75% on day 2^nd^ and 1.36% on day 3^rd^. This signifies resolving of inflammatory response in KT and hence more comfort for the patient postoperatively. Overall swelling resolves by day 7^th^ in both the groups. The postoperative mouth opening was more from the 2^nd^ day in KT group with mean of 26.07 mm and 20.33 mm in No KT group (*p* < 0.01). Trismus when noted on the 3^th^ day was also reduced with mean interincisal mouth opening of 29.13 mm in KT group and 23.20 mm when compared to the No KT group. The recovery from trismus is directly related with reduction of pain and swelling from post-operative day 2^nd^ which is seen in patients and also verified by our results.

Surgical removal of 3^rd^molars inflicts a certain degree of trauma to both hard and soft tissues of oral cavity. This initiates an inflammatory reaction involving the damaged tissues [6, 7]. Oral muscle function is not exclusively limited to the tissue movements, yet additionally controls the course of venous and lymph streams, internal temperature, and so forth. Hence, with muscle injury during 3^rd^molar surgery, various clinical manifestations are observed [4, 8]. Kinesio taping aims to provide a free range of motion to the muscles in order to ensure adequate healing biomechanically rather than restricting and immobilizing the affected muscles and joints like conventional athletic tapes [9, 10].

A similar study was conducted by Yurttutan et. al. (2020) [11] where 30 patients were randomly divided into 2 groups, half with KT application and half without it. Tape was applied directly after impacted third molar surgery and maintained for postoperative 7 days. Pain and analgesic usage were recorded on the post-op 1st, 2nd, 3rd, and 7th days. There was significantly lower use of analgesics in the group with KT application.

The results of our study are in concurrence with the study performed by Ana Carolina Heras et al. (2019) [1] in which they concluded that application of KT tape post third molar extraction reduces edema and pain intensity. In their study thirteen individuals were subjected to extraction of third molar on both sides showed positive results on the KT side 5 days after surgery.

As has been proven in the past, post operative mouth opening is directly related to associated pain and swelling. This was also confirmed in our study statistically. The recovery when observed on the seventh day was also significantly higher when compared to the No KT group. The study by Yushan Wang et. al. (2021) [12] was aimed at evaluating whether Kinesio taping (KT) can improve patient discomfort after mandibular third molar surgery. The results showed postoperative application of KT improved restricted mouth opening in the early and late postoperative periods.

The therapeutic application of KT in maxillofacial surgery has recently emerged. Furthermore, KT has been used not only for third molar surgery but also in the treatment of cases with trauma and temporomandibular disorders and those requiring orthognathic surgery [13]. In a previous study, a randomized clinical trial, by Ristow et al. (2013) [14] application of KT following surgical management of mandibular fracture and reported that it had a significant effect on tissue reaction and swelling thereby decreased the prevalence of swelling by more than 60% during the first two post-operatively.

In a recent study by Kim MG, Kim MY (2020) [15], KT application was done for management of post-operative complications after cyst enucleation. KT was applied after surgery in addition to basic postoperative care, and a control group, where patients received basic postoperative care without KT application showed that KT can effectively manage facial swelling.

Kinesio Taping is a treatment modality based on body’s own natural healing mechanism. This technique displays its adequacy through the neurological and circulatory function of the body, essentially coming from the study of Kinesiology, perceiving the significance of body and muscle actions in recovery and everyday wear and tear. It is a newer approach to treat nerves, muscles, and organs, having found its way in orthopedic treatment [12, 16].

The Kinesiologic tape is designed to have a longitudinal stretch that is comparable to human skin, stretching 55–60% of its resting length. Its thickness is similar to the epidermis of the skin to limit the body's perception of weight and avoid sensory stimuli when applied properly. Patients do not perceive any sense of tape applied to the skin after 10 min [17, 18]. Skin problems are constant issues that emerge because of exorbitant wearing of kinesiology tape. This might emerge especially because of sweat, which is an important part day to day activity. Body’s adaptation for stimulation is another fundamental factor created by kinesio tapes. Hence, for effective skin stimulation, it is required to change or reapply kinesio tape every day. In the present study, patients were advised to change KT every morning [19]. In this study, the Fan strip application method for lymphatic correction was used, with none to very light tension added to the strip tails. The tape is applied to a stretched site, creating convolutions as the skin is lifted. This reduces pressure and opens initial lymphatic channels, while the tape also generates a massaging action during active motion, improving the efficiency of deeper lymphatics by allowing maximum contraction and relaxation of muscles [17, 20].

In our study, we found that KT was effective in reducing post-operative pain, edema and trismus, particularly on second and third post-operative days. All of these complications can be minimalized with clinical therapeutic application of Kinesio tape on impacted third molar surgeries. The parameters included in the study, Pain, swelling and trismus are not independent variables. Hence, it cannot be directly related to application of KT and this was one of the major drawbacks of this study. No patient complaint of skin discomfort, pertaining to application of KT, however some complained of noticeable taping on face for 7 days, resulting in reluctance to go out in public places. This is one of the drawbacks of use of KT in maxillofacial region. Further studies are needed in well-defined patient populations.

## Conclusion

As kinesiologic taping originates from sports medicine, but can also used therapeutically for reducing post operative sequelae as demonstrated in our study. At present there are very limited evidence- based scientific studies for exploring KT application for treatment of post-operative edema in head and neck surgeries. Whereas comparing KT application with the use of analgesics is controversial. However, It may also reduce postoperative need for NSAID and analgesics, thus reducing the side effects associated with these drugs.

## Data Availability

The datasets generated and/or analysed during the current study are available in the [Zenodo] repository, [https://doi.org/10.5281/zenodo.7827614].
